# Occupational health risks among the workers employed in leather tanneries at Kanpur

**DOI:** 10.4103/0019-5278.44695

**Published:** 2008-12

**Authors:** Subodh Kumar Rastogi, Amit Pandey, Sachin Tripathi

**Affiliations:** Epidemiology Division, Indian Institute of Toxicology Research, Post Box No. 80, Mahatma Gandhi Marg, Uttar Pradesh, India; 1 Cardiovascular Toxicology, Indian Institute of Toxicology Research, Post Box No. 80, Mahatma Gandhi Marg, Uttar Pradesh, India; 2 Biochemistry, Hind Medical College, Lucknow - 226 001, Uttar Pradesh, India

**Keywords:** Chromium, health risk, leather tanneries, morbidity

## Abstract

In a cross-sectional study, a random sample of 197 male workers drawn from different sections of 10 leather tanneries in Kanpur were selected for the assessment of health risks. A control group comprising of 117 male subjects belonging to a similar age group and socioeconomic strata, who never had any occupational exposure in the leather tanneries, were also examined for the comparison purpose. The findings revealed a significantly higher prevalence of morbidity among the exposed workers in contrast to that observed in the controls (40.1% vs. 19.6%). The respiratory diseases (16.7%) were mainly responsible for a higher morbidity among the exposed workers whereas the gastrointestinal tract problems were predominant in the control group. The urinary and blood samples collected from the exposed group showed significantly higher levels of chromium, thereby reflecting the body burden of Cr in the exposed workers as a result of a high concentration of environmental Cr at the work place.

## INTRODUCTION

Kanpur is a major leather-processing centre in Uttar Pradesh in North India, with an estimated 20,000 tannery workers. Leather production includes many operations with different exposures, which can be harmful for the health of the workers, and particularly be carcinogenic.[1–3] Some compounds in the tanning process are considered as probably being carcinogenic to humans (some benzene-based dyes and formaldehyde).[4–5] Besides these, scores of other chemicals and organic solvents such as chromate and bichromate salts, aniline, butyl acetate, ethanol, benzene, toluene, sulfuric acid and ammonium hydrogen sulfide are used in the tannery industry. An important health risk factor for the tannery workers is occupational exposure to chromium, mainly in the organic Cr (Ш) form or in the protein bound-form (leather dust). Chromium may enter the body by inhalation, ingestion and by direct cutaneous contact. Professional exposure to Cr (Ш) increases the risk of dermatitis, ulcers and perforation of the nasal septum and respiratory illnesses as well as increased lung and nasal cancers.[6–12] Cr-specific health hazards like carcinoma of the larynx and lung parenchyma and paranasal sinuses have also been reported.[13–18]

The purpose of the study was, therefore, to investigate the adverse health effects of exposure to basic tanning pigments (both trivalent and hexavalent chromium salts), organic solvents and other chemicals used in the leather tanning industries at Kanpur.

## MATERIALS AND METHODS

In a cross-sectional study, a random sample of 197 male workers drawn from different sections of 10 leather tanneries in Kanpur was selected for the health survey. A reference group of 117 male subjects belonging to a similar age group and socioeconomic strata, who never had any occupational exposure in any process in the tanneries, served as a control. Most of the control subjects were self employed in petty shops and thus having no exposure to the toxicants prevailing in the tannery work environment.

The following parameters were studied:

1. Personal and occupational history
  The personal history included details of personal characteristics, age, smoking history, alcohol and tobacco consumption and family history, socioeconomic status and living conditions whereas the occupational history included details of job, duration and type of exposure and details of toxicants at the work place.
2. Clinical examination
  A detailed physical examination of the nervous, respiratory, cardiovascular, ocular, dermal and musculoskeletal system was conducted at a health examination camp set up at the work place in the tannery industry.
3. X-ray chest
  The clinically positive respiratory cases were subjected to a chest X-ray (PA view) on a 100 MA X-ray machine to confirm the clinical findings.
4. Lung function testing
  A precalibrated portable computerized spirometer (Auto spiror Model HS-1, Tuda aptics, Japan) was used to record the spirometric functions. The observed values were compared with the predicted values of Rastogi et al.^19^ The peak expiratory flow rate was measured by a peak flow meter (standard model –clement clake, U.K) and the highest value was taken into account.
5. Biomonitoring of chromium in blood and urine samples
  Venous blood samples were collected in heparinized tubes and spot urine samples in sterile plastic containers. The samples were transported to the laboratory under chilled conditions. Urinary and blood chromium analysis was performed by a direct dilution method. An atomic absorption spectrophotometer (Parkin Elmar model 5000, USA) was used for the estimation of chromium.[20]
6. Air monitoring of chromium at the work place
  External exposure at the work place was estimated by personal air monitoring during the work shift. The airborne particulate sampling was performed on quartz microfiber filters (Whatman QM-A, diameter 37mm) in Millipore filter holders. The concentrations of the total particulate were determined by weighing the filter and calculated in milligrams per cubic meter.

## RESULTS

The details of the processes and chemicals used in the leather tanning industry are listed in Table 1. The physical characteristics of the exposed group and the controls are shown in Table 2. The mean age, height and body weight of the exposed group were found to be similar to those measured in the control group. The leather tanners had a mean exposure of 8.8 + 3.20 years in the leather tanneries. The control group showed a higher prevalence (47.8%) of smoking in contrast to 42.1% observed in the tannery workers. The prevalence of respiratory symptoms was found to be significantly higher than that reported by the control group (16.7% vs. 4.27%) (*P* < 0.001). Dry/productive cough and throat irritation were the cardinal respiratory symptoms recorded in the exposed workers [Table 3].

**Table 1:** Processes and chemicals used in leather production

Processes	Chemicals used	Purpose
Preparation of the hide for tanning	DDT, zinc chloride, phenols, formaldehyde, mineral oil, arsenious anhydride	Hides are treated for defestation and disinfection
Tanning process	Calcium hydroxide, sodium sulfide, sulfuric acid, formic acid, hydrogen sulfide and solvents such as benzene, ethanol, tetrachloride, trichloroethylene and dichloromethane	For conversion of hides and skins to leather by removing the epidermis and subcutaneous layer and subsequently stabilizing the middle portion of the skin
Finishing process	Formaldehyde as fixer, aniline and resins	Includes coloring and producing surface effects to ensure brightness, softness, elasticity and impermeability
1. Casein finishing				
2. Nitrocellulose finishing.		

**Table 2:** Physical characteristics and smoking prevalence among the leather tanners and controls

Parameters	Exposed (n = 197) (mean, SD)	Controls (n = 117) (mean, SD)
Age (years)	35.5 + 10	38.6 + 11
Height (cm)	164.5 + 4.82	165.8 + 5.20
Weight (kg)	54.6 + 6.8	57.2 + 5.9
Exposed period (years)	8.8 + 3.20	–
Smoking prevalence (%)	83 + 42.1	56 + 47.8

**Table 3:** Respiratory symptoms observed among the exposed and the control group

Respiratory symptoms	Controls (n = 117) (N, %)	Exposed (n = 197) (N, %)
Dry/productive cough	2 1.7	11 5.58
Throat irritation	1 0.85	7 3.55
Chest congestion	1 0.85	6 3.04
Nasal catarrh	1 0.85	5 2.53
Dyspnoea	–	4 2.03
Overall	5 4.27	33 16.7

The overall prevalence of morbidity was found to be significantly higher among the tanners in contrast to that found in the controls (40.1% vs. 19.6%) (*P* < 0.01). In the case of the exposed group, it is the respiratory (16.7%), followed by ocular illnesses (14.7%) that afflicted the tanners, whereas gastrointestinal tract problems were predominant in the control groups (9.4%) [Table 4]. Among the leather tanners, a group of 10 subjects (5.07%) reported redness in the conjunctiva with prominent bulbar vessels, seven subjects (3.55%) reported congestion of the nasal mucosa.

**Table 4:** Type of morbidity observed in the exposed and in the control group

Morbidity	Controls (n = 117) (N, %)	Exposed (n = 197) (N, %)
Ocular	3 2.56	29 14.7
Dermal	4 3.41	17 8.6
Respiratory	5 4.27	33 16.7
Gastrointestinal	11 9.40	–
Overall	23 19.6	79 40.1

Nearly 9% (n = 17) of the leather tanners reported dermatological diseases such as rashes and papules along with complaints of itching. The burning sensation was reported by 15 subjects (7.6%) in the exposed workers.

Among the respiratory morbidity in the exposed group, it was occupational asthma that showed a prevalence of 5.0% whereas chronic bronchitis, allergic bronchitis, sinu sites and pulmonary tuberculosis were other respiratory illnesses observed in the tanners. No cases of asthma and allergic bronchitis were reported by the control group [Table 5].

**Table 5:** Respiratory morbidity of the leather tanners and controls

Respiratory morbidity	Controls (n = 117) (N, %)	Exposed (n = 197) (N, %)
Chronic bronchitis	1 0.85	4 2.03
Occupational asthma	–	10 5.07
Allergic bronchitis	–	7 3.55
Sinusitis	2 1.7	7 3.55
Pulmonary tuberculosis	1 0.85	5 2.53
Overall	4 3.41	33 16.7

All lung function values were significantly reduced in the exposed workers. The reduced values indicated bronchial obstruction in the exposed group [Table 6].

**Table 6:** Lung function values among the exposed and the control groups

Parameters	Controls (n = 117) (mean, SD)	Exposed (n = 197) (mean, SD)
FVC (%P)	81.4 ± 7.4	76.8 ± 7.8
FEV (%)	83.6 ± 8.6	70.6 ± 6.8
FEV/FVC (%)	79.8 ± 10.4	68.6 ± 9.7
FMF (1/s)	3.42 ± 0.67	2.09 ± 0.65
PERF (%P)	450 ± 60.8	285 ± 40.8

The prevalence of respiratory impairment was found to be significantly higher among the leather tanners in contrast to that observed in the control group (30.9% vs. 16.2%) (*P* < 0.001). Bronchial obstruction followed by lung restriction was the main abnormality found in the exposed group [Table 7].

**Table 7:** Type of pulmonary impairment among the tannery workers and the controls

Type of pulmonary impairment	Exposed (197, %)	Controls (17. %)
Bronchial obstruction	29 14.7	7 5.98
Lung restriction	17 8.6	5 4.27
Mixed ventilator defects	15 7.6	7 5.98
Overall	61 30.9	19 16.2

The urinary and blood concentrations of Cr were found to be significantly raised among the leather tanners (*P* < 0.001), thereby reflecting the body burden of Cr in the exposed workers as a result of a high concentration of environmental Cr at the work place [Table 8].

**Table 8:** Urinary and blood chromium levels in the exposed and in the control groups

Parameters	Controls (n = 21)	Exposed (n = 39)	*P*-value
Urinary Cr (ppb)	1.37 ± 0.49	(0.67 – 2.17)	0.001
Mean ± SD	5.39 ± 2.19	(2.59 – 11.81)	
Range			
Blood Cr (ppb)	1.68 ± 0.41	(0.57 – 2.15)	0.001
Mean ±SD	2.62 ± 0.57	(1.46 – 4.95)	
Range			

## DISCUSSION

An important health risk factor for the tannery workers is occupational exposure to chromium, which is used as a basic tanning pigment. The workers on exposure to leather dust, which contains chromium in the protein-bound form, exhibited a higher mean concentration of urinary and blood chromium than the reference values. The personal sampling conducted at different work sites exhibited higher levels of total chromium. The high morbidity (40.1%) observed in the tanners in comparison to the reference values (19.6%) could be due to high levels of chromium in the biological samples of the exposed workers and air samples collected at the worksite. The increased morbidity in the exposed workers could be attributed to high respiratory illness (16.7%) compared with 4.27 % in the control group. The higher biological values of chromium among the tanners could be explained by atmospheric pollution caused by the liberated leather dust at the work place. The increased pulmonary morbidity is also associated with certain characteristic symptoms such as dry cough (5.6%), throat irritation (3.6%) and lung congestion in 3.0% of the workers. Among the respiratory morbidity in the exposed group, the cases of occupational asthma (5.0%) were more prominent than other respiratory illnesses. It has been reported in the literature that it is the metabolism distribution and transport of the chromium in the blood that is a causal factor for increased respiratory morbidity.[21,22] The hexavalent chromium is rapidly absorbed by the lungs into the blood and easily penetrates the cellular membranes and binds to the hemoglobin in the red blood cells thereby affecting the oxygen carrying capacity and impairing the lung function status.[23–25]

Our study confirms the previous findings of increased pulmonary impairment among the chrome platers (30.9%) as against 16.2% in the controls.[26] In an earlier study on chrome platers, we demonstrated cases of nasal ulcer and nasal septum perforation.[27] However, in the present study, we did not find any case of nasal ulcer and perforation. Our study confirms the earlier reports that the biological levels of chromium (in urine and blood) are good indicators of the exposure level of chromium at the work place.[28–30] Thus, air and biological monitoring can effectively be used for assessing the external and internal exposure to chromium and associated health risks among the exposed human population in epidemiological health surveys.[20]

## CONCLUSION

The high morbidity among the tannery workers may be due to elevated levels of urinary and blood chromium levels resulting from increased air levels of chromium at the work place. The study recommends that the biomonitoring of the chromium levels in the biological fluids can serve as a useful tool for mitigating the health hazards and risk factors in the exposed workers.

## References

[1] Stern FB (2003). Mortality among chrome leather tannery workers: An update. Am J Ind Med.

[2] Issever H, Ozdilli K, Ozyildirim BA, Hapcioglu B, Ince N, Ince H (2007). Respiratory problems in tannery workers in Istanbul. Indoor Built Environ.

[3] Kornhauser C, Katarzyna W, Kazimierz W, Malacara JM, Laura EN, Gomez L (2002). Possible adverse effect of chromium in occupational exposure of tannery workers. Industrial Health.

[4] International Agency for Research on Cancer (IARC) (1987). Overall evaluation of carcinogenicity: An updating of IARC Monograph. IARC Monograph on the evaluation on the carcinogenicity risk of chemicals to human.

[5] Budhwar R, Das M, Bihari V, Kumar S (2005). Exposure estimates of chrome platters in India:an exploratory study. Biomarkers.

[6] Stern RM, Berlin A, Fletcher A (1986). International conference on health hazards and biological effects of welding fumes and gases: Copenhagen 18-21, February, 1985. Int Arch Occup Environ Health.

[7] Angerer J, Amin W, Heinnrich-Ramm R (1987). Occupational chronic exposure to metals. Arch Occupational Environ Health.

[8] Lin SC, Tai CC (1994). Nasal septum lesion caused by chromium among chromium electroplating workers. Am J Ind Med.

[9] Stern AH, Bragt PC (1993). Risk assessment of the allergic dermatitis: Potential of environmental exposure to hexavalent chromium. J Toxicol Environ Health.

[10] Mikoczy Z, Hagmar L (2005). Cancer incidence in the Swedish leather tanning industry: Updated findings 1958-99. Occup Environ Med.

[11] Veyalkin LV, Milyutin AA (2003). Proportionate cancer mortality among workers in the Belarussian tanning industry. Am J Ind Med.

[12] Basketter D, Horev L, Slodovnik D, Merimes S, Trattner A, Ingber A (2001). Investigation of the threshold for allergic reactivity to chromium. Contact Dermatitis.

[13] Walsh EN (1953). Chromate hazards in industry. J Am Med Assoc.

[14] Browning E (1975). Toxicity of industrial metals.

[15] Royale H (1975). Toxicity of chromic acid in chromium plating industry. Environ Res.

[16] Langardt S (1990). One hundred years of chromium and cancer: A review of epidemiological evidence and selected case reports. Am J Ind Med.

[17] Mikoczy Z, Hagmar L (2005). Cancer incidence in the Swedish leather tanning industry: Updated findings. Occup Environ Med.

[18] Rastogi SK, Kesavchandran C, Mahdi F, Panday A (2007). Occupational cancers in leather tanning industries: A short review. Indian J Occup Environ Med.

[19] Rastogi SK, Mathur N, Clerk SH (1983). Ventilatory norms in healthy industrial workers. Ind J Chest Allied Sci.

[20] Schermaier AJ, O'Conner LH, Pearson KH (1985). Semi automated determination of chromium in whole blood in serum by Zeeman electro thermal atomic absorption spectrophotometery. Clin Chim Acta.

[21] Langardt S, Langardt S (1982). Absorption, transport and excretion of chromium in man and animals. Biological and environmental aspects of chromium.

[22] Agency for Toxic Substances and Disease Registry. Toxicological Profilefor Chromium.

[23] Mutti A, Cavatorta A, Pedroni C, Borghi A, Giaroli C, Franchini I (1989). The role of chromium accumulation in the relationship between airborne and urinary chromium in welders. Int Arch Occup Environ Health.

[24] Morris BW, Griffiths H, Hardisty CA, Kemp GJ (1989). Increased concentrations of chromium in plasma, urine and red blood cells in a group of stainless steel workers. Atom Spectrosc.

[25] Benova D, Hadjidekova V, Hristova R, Nikolova T, Boulanova M, Georgieva I (2002). Cytogenetic effects of hexavalent chromium in Bulgarian chromium platers. Mutat Res.

[26] Das M, Kumar P, Kumar A (1989). Lung function studies among chrome platers. Indian J Occup Health.

[27] Rastogi SK (1992). Epidemiological health survey of workers engaged in Lock manufacturing industry in Aligarh.

[28] Sjogren B, Hedstrom L, Ulfvarson U (1983). Urine chromium as an estimator of air exposure to stainless steel welding fumes. Int Arch Occup Environ Health.

[29] Tola S, Kilpio J, Virtzmo M, Happa K (1977). Urinary chromium aqs an indicator of the exposure of welders to chromium. Scand J Work Environ Health.

[30] Rahkonen E, Junttila ML, Kalliomäki PL, Olkinouora M, Koponen M, Kalliomäki K (1983). Evaluation of biological monitoring among stainless steel workers. Int Arch Occup Environ Health.

